# Visual Evoked Potential and Magnetic Resonance Imaging are More Effective Markers of Multiple Sclerosis Progression than Laser Polarimetry with Variable Corneal Compensation

**DOI:** 10.3389/fnhum.2014.00010

**Published:** 2014-01-22

**Authors:** Ema Kantorová, Peter Žiak, Egon Kurča, Mária Koyšová, Mária Hladká, Kamil Zeleňák, Jozef Michalik

**Affiliations:** ^1^Neurology Clinic, Jessenius Faculty of Medicine, Comenius University in Bratislava, Martin, Slovakia; ^2^Ophthalmology Clinic, Jessenius Faculty of Medicine, Comenius University in Bratislava, Martin, Slovakia; ^3^Radiology Clinic, Jessenius Faculty of Medicine, Comenius University in Bratislava, Martin, Slovakia

**Keywords:** VEP, laser polarimetry of optic nerve, MRI, multiple sclerosis, demyelinization and axonal degeneration

## Abstract

**Background:** The aim of our study was to assess the role of laser polarimetry and visual evoked potentials (VEP) as potential biomarkers of disease progression in multiple sclerosis (MS).

**Participants:** A total of 41 patients with MS (82 eyes) and 22 age-related healthy volunteers (44 eyes) completed the study. MS patients were divided into two groups, one (ON) with a history of optic neuritis (17 patients, 34 eyes) and another group (NON) without it (24 patients, 48 eyes). The MS patients and controls underwent laser polarimetry (GDx) examination of the retinal nerve fiber layer (RNFL). In the MS group, we also examined: Kurtzke “expanded disability status scale” (EDSS), the duration of the disorder, VEP – latency and amplitude, and conventional brain magnetic resonance imaging (MRI). Our results were statistically analyzed using ANOVA, Mann–Whitney, and Spearman correlation analyses.

**Results:** In the MS group, brain atrophy and new T2 brain lesions in MRI correlated with both VEP latencies and amplitudes. Separate comparisons revealed VEP latency testing to be less sensitive in ON than in NON-patients. In ON patients, VEP amplitudes correlated mildly with brain atrophy (*r* = −0.15) and strongly with brain new MRI lesions (*r* = −0.8). In NON-patients, highly significant correlation of new MRI brain lesions with VEP latencies (*r* = 0.63, *r* = 0.6) and amplitudes (*r* = −0.3, *r* = −4.2) was found. EDSS also correlated with brain atrophy in this group (*r* = 0.5). Our study did not find a correlation of GDx measures with MRI tests. The GDx method was not able to detect whole brain demyelinization and the degeneration process, but was only able to reveal the involvement of optic nerves in ON and NON-patients.

**Conclusion:** In our study, we found that both methods (VEP and GDx) can be used for the detection of optic nerve damage, but VEP was found to be superior in evaluating whole brain demyelinization and axonal degeneration. Both VEP and MRI, but not GDx, have an important role in monitoring disease progression in MS patients, independent of the ON history.

## Introduction

Multiple sclerosis (MS) is characterized as a progressive inflammatory autoimmune disease of the central nervous system in which axonal destruction occurs at early stages and is responsible for the accumulation of irreversible disability (Kuhlmann et al., 2002).

The proposed mechanisms of axonal degeneration in MS are considered to vary depending on the stage of the disease. During the early stages of disorder, axonal injury correlates with the magnitude of the inflammation. It has been proposed that in the later stages, accumulation of tissue damage and loss of remyelination cause irreversible disability (Kuhlmann et al., 2002). Defining the subclinical progression of the disorder is very challenging. Standard clinical end points such as expanded disability status scale (EDSS) provide an indication of MS activity, but do not always reveal early neurodegeneration changes. In recent years, axonal damage and disease progression markers have been tested for use in monitoring the early subclinical changes, since existing methods have not been proved sensitive enough in evaluating the disorder. In the future, short-lasting, highly available, and easy-performed methods are expected to be available. In our study, we focused on ophthalmological and electrophysiological tests. Scanning laser polarimetry (GDx) with variable corneal compensation (VCC) is a non-invasive imaging technique based on the projection of polarized near-infrared light and its retardation, when propagating through a birefringent medium – i.e., retinal nerve fiber layer (RNFL). It can detect changes resulting from axonal cytoskeleton disorganization. GDx measures both the thickness and integrity of RNFL. The potential of GDx to measure the RNFL is considered to be comparable to that of optic coherence tomography (OCT) (Zaveri et al., 2008; Galleta and Balcer, 2011).

Electrophysiological tests, including visual evoked potentials (VEP), are among the newer approaches to examination that are to be further investigated. Nowadays, they are considered to be complementary to the clinical measurement of somatosensory functions. VEP plays a useful role in the detection of early subclinical changes of nerve tract demyelinization. It may have a role similar to structural measures in detecting, and hence monitoring the signs of early axonal destruction.

Here, we compare VEP and GDx with conventional magnetic resonance imaging (MRI) to determine which of these markers is most suitable to detect whole brain demyelinization and axonal degeneration in MS.

## Subjects and Methods

### Patients

Patients with MS and disease-free patients (controls) participated in the study of the visual function and the neuronal integrity of the RNFL. MS patients were divided into two groups, one (ON) with history of optic neuritis (17 patients, 34 eyes) and another group (NON) without it (24 patients, 48 eyes). A total of 45 patients with MS were recruited from patients treated in the MS Centre at University Hospital in Martin, Slovakia. The inclusion criterion for enrollment of ON patients was the history of optic neuritis for at least 3 months prior to the date of study entry. Four of them were excluded from the group: one patient had an amblyopic eye, three had moderate to severe loss of visual acuity and difficulty concentrating during the ophthalmologic examination. The remaining 17 ON patients were enrolled. NON-patients were randomly selected from the patients of the MS centre. Out of the enrolled MS patients, 90% (*n* = 37) had a relapse-remitting course of the disorder, two had a secondary progressive course, and two were diagnosed as having a clinically isolated syndrome. At the time of the examination, the patients were undergoing treatment by immunomodulatory agents (interferon beta, glatiramer acetate, or intravenous methylprednisolone). These treatments complied with the authorized recommendations for MS treatment in the Slovak Republic. The mean length of treatment at the time of entry into the study was 4.01 ± 2.8 years (*p* = 0.14 between ON and NON).

The control group consisted of 22 age-related disease-free persons with no history of visual problems and no evidence of demyelinization disorder. Table 1 describes the demographic and clinical characteristics of the patients.

**Table 1:** **Demographic and clinical characteristics of the patients**.

	ON	NON	Controls
Patients/eyes	17/34	24/48	22/44
Females	14	14	17
Age	34.0 ± 10.6	37.6 ± 9.3°	39.8 ± 11.5°°
Disease duration	6.5 ± 1.1	5.8 ± 0.96*	–
EDSS	2.6 ± 1.1	2.4 ± 1.1	–

*°The difference between ON and NON-groups *p* = 0.16*.

*°°The difference between Con and MS *p* = 0.12*.

*The difference between groups *p* = 0.33 (*p* = 0.05 statistical significance), ANOVA test.

*ON = patients with history of ON, NON = patients without history of ON*.

*EDSS = expanded disability status scale*.

### Clinical and paraclinical examinations

#### Patients

Routine neurological and neurophysiological examinations were performed on patients in the MS group. Complete visual function tests were supplemented with GDx analysis. Optic neuritis was confirmed by ophthalmological examination including a visual acuity test, a color vision test, and fundoscopic examination of the retina and the optic disk. Episodes of optic neuritis were found to have occurred with the same frequency in both left and right eyes.

The duration of disease was recorded in all participants. EDSS and ophthalmology testing were performed at the same sessions. Conventional MRI 1.5 T scans had been performed annually since the date of diagnosis. At the time of study entry, new MRI scans were performed to evaluate the range of brain atrophy. T2, T1, Flair, and proton density-weighted imaging (PD-weighted images) were used. The measurement of brain atrophy (level of neurodegeneration) was performed with semi-automated radiological protocol based on the manual segmentation technique. Semi-automated edge detection contouring–thresholding technique was manually corrected for the region of interest. The progression of brain volume loss was detected after comparison with previously measured values. Brain atrophy was determined longitudinally by serial MRI in each individual patient. The final results were affirmed by a radiologist either as positive (presence of brain atrophy) or negative (brain atrophy not found). Comparison of new T2 hyperintense lesions on follow-up scans with the baseline measurements provided evidence for the intensity of inflammation. The presence of two or more new lesions in the current T2-weighted MRI was interpreted as a positive sign of active inflammation. MRI measurements were made at the same time as VEP and GDx and evidence of lesions.

A Myoquick ISA 1800 EP from Micromed was used for VEP P100 waves examination. Latency and amplitude of P100 waves were individually judged.

The control group of 22 age-related disease-free subjects underwent ophthalmological tests.

### Retinal nerve fiber layer measurement

Patients with MS and controls underwent measurement of the RNFL thickness for both eyes using GDx with VCC (software version 5.5.1, 2005 Carl Zeiss Meditec). These scans were centered on the optic disk using a scan circle of 3.2 mm; the mean of three separate measurements was used. The temporal–superior–nasal–inferior–temporal average RNFL thickness was used as the summary parameter for GDx. RNFL pathology was assessed by an ophthalmologist trained in GDx analysis.

### Statistical analysis

Analyses were performed using NCSS – Statistical and Power Analysis Software 2007 and STAT. Differences across all groups were assessed using ANOVA tests, which were applied for the determination of differences between the data of the patient and control groups. The relationship between healthy and MS eyes was examined by Mann–Whitney test, separately for both ON and NON-groups. Spearman correlation analysis was used to determine the relationship of MRI measures with VEP parameters, RNFL thickness, EDSS, and disease duration. Statistical methods were consulted with Peter Slezak from Bio-Med-Stat Center (Faculty of Mathematics, Physics, and Informatics, Comenius University in Bratislava).

## Results

In the ON group, both brain atrophy and MRI brain new lesions correlated with lower VEP amplitudes. This correlation was found only in the right eye. We did not find any significant association of VEP latency and brain atrophy or new brain lesions in MRI. Both disease duration and age of patients significantly correlated with brain atrophy measures, but we did not find this relationship with new MRI brain lesions. EDSS score was also related to brain atrophy but not to new MRI brain lesions. The results are shown in Table 2A.

**Table 2:** **(A) Correlation of MRI with age, VEP, RNFL, EDSS and disease duration ON patients. (B) Correlation of MRI with age, VEP, RNFL, EDSS and disease duration NON-patients**.

(A)		

ON patients (*n* = 17)	MRI brain new lesions	MRI brain atrophy
Age (years)	ns	*r* = 0.3, *p* = 0.09
VEP lat right (ms)	ns	ns
VEP ampl right (μV)	*r* = −0.8, *p* = 0.0002	*r* = −0.15, *p* = 0.07
VEP lat left (ms)	ns	ns
VEP ampl left (μV)	ns	ns
RNFL right (μm)	ns	ns
RNFL left (μm)	ns	ns
EDSS (1–10)	ns	*r* = 0.42, *p* = 0.006
Disease duration (years)	ns	*r* = 0.5, *p* = 0.02

**(B)**		

**NON-patients (*n* = 24)**	**MRI brain new lesions**	**MRI brain atrophy**

Age (years)	*r* = 0.46, *p* = 0.02	ns
VEP lat right	*r* = 0.36, *p* = 0.08	*r* = 0.63, *p* = 0.001
VEP ampl right	*r* = −0.3, *p* = 0.09	*r* = −0.1, *p* = 0.09
VEP lat left	*r* = 0.4, *p* = 0.03	*r* = 0.6, *p* = 0.01
VEP ampl left	*r* = −0.42, *p* = 0.04	*r* = −0.5, *p* = 0.02
RNFL right	ns	ns
RNFL left	ns	*r* = −0.4, *p* = 0.05
EDSS	*r* = 0.3, *p* = 0.09	*r* = 0.5, *p* = 0.01
Disease duration	ns	*r* = 0.5, *p* = 0.09

*Spearman analysis (*p* = 0.05 statistical significance), ns = non-significant, MRI brain atrophy = presence of brain atrophy, MRI brain new lesions = T2 hyperintense new brain lesions, EDSS = expanded disability status scale*.

In patients without a history of ON (NON), VEP parameters showed even stronger association with both MRI new brain lesions and brain atrophy than in ON group. We found a significant but moderate relationship of new brain T2 lesions with longer latencies of VEP in the left and right eyes. We also found correlation of VEP amplitudes and MRI new brain lesions. VEP latency delay correlated significantly with duration of disorder. EDSS values correlated with brain atrophy and also mildly with MRI new brain lesions. In this group, association of RNFL thickness and brain atrophy was proved for the left eye. MRI brain atrophy correlated with disease duration. Results are shown in Table 2B.

Multiple sclerosis patients from both groups did not differ in many parameters such as age (*p* = 0.12), duration of disorder (*p* = 0.32), duration of treatment (*p* = 0.12), EDSS (*p* = 0.32), VEP latency right (*p* = 0.3), VEP amplitude right (*p* = 0.67), VEP amplitude left (*p* = 0.7), RNFL thickness right (*p* = 0.6) and left (*p* = 0.3), and nerve fiber indicator (NFI) right (*p* = 0.97) and NFI left (*p* = 0.3). The only difference found was in VEP latency in left eyes (*p* = 0.01). This fact encouraged us to evaluate both MS groups together.

Both brain atrophy and new MRI brain lesions have shown stronger correlation with VEP results than with GDx RNFL measures.

Brain atrophy correlated with VEP latencies of the right eyes of the MS patients, but not of the left ones. A significant correlation of VEP latencies and VEP amplitudes with new brain lesions in MRI was found in right and left eyes. The results are described in Tables 3A,B.

**Table 3:** **(A) Correlation of VEP latency and MRI brain atrophy, MRI brain new lesions in all MS patients; (B) correlation of VEP amplitudes and MRI brain atrophy, MRI brain new lesions in all MS patients**.

(A)		

	VEP lat right/MRI correlation	VEP lat left/MRI correlation
MRI atrophy	*r* = 0.63, *p* = 0.01	ns
MRI brain new lesions	*r* = 0.6, *p* = 0.02	*r* = 0.6, *p* = 0.03

**(B)**		

	**VEP ampl right/MRI correlation**	**VEP ampl left/MRI correlation**

MRI atrophy	*r* = −0.4, *p* = 0.03	*r* = −0.5, *p* = 0.08
MRI brain new lesions	*r* = −0.7, *p* = 0.004	*r* = −0.6, *p* = 0.01

*Spearman correlation test (*p* = 0.05 statistical significance). VEP lat = latency of visual evoked potential, VEP ampl = amplitude of visual evoked potential, MRI atrophy = presence of brain atrophy, MRI brain new lesions = T2 hyperintense new brain lesions*.

A relationship was observed in VEP latencies and EDSS scores in right and left eyes. Amplitudes of VEP did not match with EDSS (Table 4).

**Table 4:** **Correlation of EDSS and VEP latency and VEP amplitude in all MS patients**.

MS patients (*n* = 41)	VEP latency right (ms)	VEP ampl right (μV)	VEP latency left (ms)	VEP ampl left (μV)
EDSS	*r* = 0.8, *p* < 0.000	ns	*r* = 0.64, *p* = 0.007	ns

*Spearman correlation test (*p* = 0.05 statistical significance). VEP lat = latency of visual evoked potential, VEP ampl = amplitude of visual evoked potential*.

When compared with healthy controls, statistical analysis showed reduction of RNFL measured by GDx of optic nerve MS eyes. The results were supported by the measurement of NFI, which serves as a comparator of normal and damaged peripapillary nerve fibers. Higher levels of NFI indicate advanced impairment of optic nerve fibers. NFI values of MS patients were higher and significantly different from values of healthy controls. Results of average RNFL thicknesses are shown in Table 5.

**Table 5:** **GDx RNFL in groups: ON, NON, and controls**.

	ON (*n* = 17)	CON (*n* = 22)	ON/CON	NON (*n* = 24)	NON/CON
RNFL right (μm)	51.0 ± 1.28 (*m* = 52.0)	57.06 ± 1.09 (*m* = 57.6)	*p* = 0.0014	52.5 ± 1.05 (m = 52.5)	*p* = 0.002
RNFL left (μm)	50.5 ± 1.36 (*m* = 50.2)	56.6 ± 1.19 (*m* = 57.35)	*p* = 0.001	53.2 ± 1.92 (m = 52.0)	*p* = 0.015
NFI right (μm)	24.1 ± 3.7 (*m* = 18.5)	15.0 ± 5.2 (*m* = 14.5)	*p* = 0.005	24.3 ± 1.4 (*m* = 21.5)	*p* = 0.05
NFI left (μm)	23.8 ± 2.9 (*m* = 25.5)	15.1 ±5.2 (*m* = 13.5)	*p* = 0.0001	21.8 ± 1.3 (*m* = 21.0)	*p* = 0.001

*Mann–Whitney test (*p* = 0.05 statistical significance), MS = multiple sclerosis, ON = history of optic neuritis, NON = without history of optic neuritis, CON = controls, RNFL = retinal nerve fiber layer, NFI = nerve fiber indicator*.

Despite the significant difference in GDx RNFL of MS and healthy eyes, neither MRI brain atrophy nor new MRI brain lesions correlated with GDx RNFL thickness and NFI in the MS group.

GDx RNFL measures correlated with VEP latencies, but not with VEP amplitudes. We did not confirm any association between RNFL thickness and disease duration.

The results are in Table 6.

**Table 6:** **Correlation of RNFL with VEP and disease duration**.

	RNFL right (μm)	RNFL left (μm)
VEP lat (ms)	*r* = −0.86, *p* = 0.001	*r* = −0.5, *p* = 0.009
VEP ampl (μV)	ns	ns
Disease duration (years)	ns	ns

*Spearman correlation test (*p* = 0.05 statistical significance), RNFL = retinal nerve fiber layer, VEP = visual evoked potential, ns = non-significant*.

## Discussion

Many studies have shown the presence of alteration of the visual system in MS as a possible method to test its subclinical progression (Sisto et al., 2005; Zaveri et al., 2008; Pueyo et al., 2010). There are different opinions as to which examination is the most appropriate. In several studies, two or more tests were performed on the same sample of patients to increase the overall sensitivity. Only a few studies have reported MRI correlations with GDx and VEP (Frohman et al., 2009).

In our study, VEP tests have been found to be more sensitive than GDx. There was a reduction in VEP amplitude in a group of patients with axonal damage (MRI brain atrophy) or active conduction block due to focal demyelinization (new T2 brain lesions in MRI), while VEP latencies remained abnormal in a group of patients with diffuse demyelinization and also with extensive axonal damage (VEP P100 correlated with MRI new T2 lesion and brain atrophy).

Separate comparisons have revealed the VEP latency testing to be less sensitive in ON than NON-patients in detecting axonal damage. In ON patients, VEP amplitude was linked to brain atrophy and also to new MRI brain lesions, but only in the right eye of subjects. We explain this by the fact that the residual axonal deficit was more pronounced in the right than in the left eye. Axonal degeneration seems to be non-equally implicated in optic nerves. In ON group, recovery from optic neuritis was less prominent in the right than in the left eye. On the other hand, positive VEP tests in NON-group have confirmed capability of VEP to reveal silent demyelinization and brain atrophy processes.

Magnetic resonance imaging was not aimed at detecting optic nerve involvement, and we did not use specific orbital short T1 inversion recovery (STIR) sequences for optic nerve evaluation. In that situation, MRI outcomes reflected intensive demyelinization and axonal degeneration of brain without determination of pre- and retrochiasmatic processes. Therefore, our results support the importance of both methods, VEP and MRI, in disease progression control.

Number of T2 lesions is considered to be a weak predictor of disability in MS (Barkhof, 1999). In NON-group of our patients, EDSS values mildly correlated with new brain MRI T2 lesions. This association reflected activity of the disorder. At the time of the examination, the patients were undergoing treatment by first line immunomodulatory agents, but many of them were shifted to another first line or second line medication in the following 12-month period (45% from NON-group). MRI is traditionally used to monitor disease progression.

Some authors have also reported the important role of VEP in the prediction of the disability progression (Trip et al., 2007; Klistorner et al., 2011). Frederiksen et al. compared the sensitivity of MRI and electrophysiological tests (VEP and somatosensory evoked potential). The combination of these methods revealed abnormalities in 63% of the patients, but only monosymptomatic subacute optic neuritis patients were tested. In this 1-year follow-up study, the mean latency of VEP in patients with optic neuritis was longer in clinically definite MS patients (Fredericsen et al., 1991).

The strong correlation of cerebrospinal fluid oligoclonal bands with VEP and multifocal MRI (*p* < 0.0000) in patients following optic neuritis reflects the activity of the demyelinization processes, as it was found by Horwitz et al. The authors proposed performing VEP and MRI examinations before deciding on a lumbar puncture, due to the high predictive role of both methods. Their patients who had normal VEP and MRI findings had a 96% probability of oligoclonal bands – negative cerebrospinal fluid values obtained by lumbar puncture samples (Horwitz et al., 2012).

Based on VEP, MRI, perimetry, and contrast sensitivity tests, Sisto et al. reported a large prevalence of visual pathway involvement in patients affected by MS with no history of optic neuritis and no visual symptoms. Despite low specificity, the tests were sensitive enough to reflect inflammation and subsequent degeneration of CNS, even in patients without history of optic neuritis. The usefulness of the combination of multiple examinations in the detection of nearly all cases of visual pathway involvement was emphasized (Sisto et al., 2005).

In our work, the predictive prognostic value of VEP has been proved by positive association of VEP latencies and EDSS in ON and NON MS patients.

Other reported data suggest that VEP and multimodal evoked potentials may be more sensitive than clinical and MRI measures in detecting disease evolution. Transversal and longitudinal studies by Leocani demonstrated good correlation between evoked potentials abnormalities and disability. Multimodal evoked potentials abnormality score correlated significantly with EDSS and MRI in transversal evaluation (Leocani et al., 2000).

We found a significant thinning of the nerve fiber layer in the retina in all patients treated for MS, compared with healthy volunteers. Other authors found similar results (Della Mea et al., 2007; Pueyo et al., 2008, 2010; Zaveri et al., 2008).

Our study did not find a significant correlation of GDx measures and MRI tests, except for a mild association of RNFL thickness of the left NON-eye with MRI brain atrophy. The GDx method was not able to detect whole brain demyelinization and degeneration processes, but revealed only the involvement of optic nerves in ON and NON-patients.

Advanced MRI techniques can provide explicit information; some studies have documented their sensitivity (Sipman et al., 2010). Frohman et al. compared non-conventional MRI techniques (magnetization transfer ratio and diffusion tensor imaging) with OCT and GDx VCC parameters. They found that the association of OCT-measured RNFL thickness with brain MRI measures was stronger than with GDx-measured RNFL thickness (Frohman et al., 2009). Zimmermann et al. (2012) referred to OCT RNFL as a parameter of neuro-axonal damage comparably linked to white and gray matter atrophy. A significant correlation between magnetization transfer ratio (MTR), measures of axonal loss by OCT-RNFL thickness, and VEP amplitude was referred by Trip et al. (2007). Whole-optic nerve MTR correlated modestly with central-field VEP latency, but strongly with lesion-only MTR measures. While latency delay correlated significantly with MTR, correlation became non-significant when adjusted for the degree of axonal loss (Trip et al., 2007; Klistorner et al., 2011). The use of non-conventional MRI techniques in monitoring patients in clinical practice is not widely advisable at the moment. All these techniques still need to be evaluated for sensitivity and specificity in detecting tissue damage in MS and its changes over time (Filippi et al., 2011). Conventional MRI is a widely available diagnostic tool in clinical practice. Our findings highlight the usefulness of conventional MRI in the evaluation of MS progression. MRI may still be a good biomarker for MS evolution.

In our MS patients, RNFL measures correlated with VEP latencies but not with VEP amplitudes.

The association of prolonged VEP latencies with RNFL thinning was also shown by Pueyo et al. (2008). Other authors have found a correlation of VEP amplitudes with RNFL thinning, or no relationship, but only under OCT results (Parisi et al., 1999). Naismith et al. considered VEP to be relatively more sensitive than OCT in detecting cases with mild to moderate visual deficit. They provided results of a retrospective study with 65 subjects with systematical evaluation of OCT and VEP in MS patients with history of optic neuritis ≥6 months prior to enrollment. They found that OCT identified RNFL thinning in 60% of eyes with previous ON, and in about 20% of subclinically affected eyes. Positive results of VEP were detected in 81% (Naismith et al., 2009). Pueyo et al. (2010) showed that GDx can be as accurate as OCT and their measurement significantly correlated with VEP P100 latencies. Correlations of GDx and VEP results were described by Della Mea et al. (2007). They found standard automated perimetry and VEP to be more sensitive to axonal defects in RNFL than GDx in MS. In contrast, a prospective study of patients with MS showed GDx to be superior to functional ophthalmological and neurophysiological tests (Garcia-Martin et al., 2010).

## Conclusion

In our study, we found that both VEP and GDx can be used as supplementary methods in detection of optic nerve damage. We confirmed the superiority of VEP over GDx in evaluating whole brain demyelinization and axonal degeneration. Both VEP and MRI, but not GDx, have an important role in monitoring disease progression in MS patients, independently of the ON history. The importance of VEP was supported by its significant correlation with MRI measures and EDSS values.

## Conflict of Interest Statement

The authors declare that the research was conducted in the absence of any commercial or financial relationships that could be construed as a potential conflict of interest.

## Abbreviations

EDSS = expanded disability status scale; GDx = laser polarimetry with variable corneal compensation; MRI = magnetic resonance imaging; MRI T1 = T1-weighted imaging; MRI T2 = T2-weighted imaging; MS = multiple sclerosis; MTR = magnetization transfer ratio; NFI = nerve fiber indicator; NON = no history of optic neuritis; OCT = optic coherence tomography; ON = optic neuritis; PD = proton density-weighted imaging; RNFL = retinal nerve fiber layer; STIR = short T1 inversion recovery.
